# Bilateral posterior shoulder dislocations with reverse Hill-Sachs lesions: a report of two cases and a literature review

**DOI:** 10.1186/s12891-026-09537-y

**Published:** 2026-01-26

**Authors:** Molin Shen, Kun Yan, Fengjie Jin, Wenzhi Zhao, Deyue Pan

**Affiliations:** Department of Orthopaedic Trauma, The Second Hospital of Dalian Medical University, No. 467, Zhongshan Road, Shahekou District, Dalian, 116023 China

**Keywords:** Posterior shoulder dislocation, Reverse Hill-Sachs lesions, McLaughlin procedure, Modified McLaughlin procedure

## Abstract

**Background:**

Posterior shoulder dislocation accompanied by reverse Hill-Sachs lesions is relatively rare, whereas bilateral posterior shoulder dislocation is exceedingly rare.

**Case presentation:**

We report two cases of bilateral posterior shoulder dislocations associated with reverse Hill-Sachs lesions. In Case 1, the lesions were managed using the McLaughlin procedure and the modified McLaughlin procedure. In Case 2, open reduction and internal fixation of the left shoulder was unsuccessful; consequently, reconstruction of the right humeral head was performed using bone cement.

**Conclusions:**

Bilateral posterior shoulder dislocations with reverse Hill-Sachs lesions are uncommon and are therefore prone to misdiagnosis. Early recognition and appropriately selected treatment strategies, tailored to the severity of the injury, are essential to achieve satisfactory functional outcomes. For severely comminuted proximal humeral fractures, when internal fixation is not feasible and no joint replacement prostheses are unavailable, bone cement reconstruction may serve as a reasonable alternative.

## Background

The shoulder joint is one of the most flexible joints in the human body and is one of the most prone to dislocation. Posterior shoulder dislocation is relatively rare. While many surgeons believe that the incidence of posterior dislocations is higher than reported in the literature, there has been little published evidence to substantiate this assumption. Posterior dislocation associated with bony impaction, specifically a compression fracture of the humeral head (reverse Hill-Sachs lesions), accounts for approximately 2–4% of shoulder dislocations [1]. The incidence of bilateral shoulder dislocations associated with reverse Hill-Sachs lesions is extremely low. Here, we report two cases of bilateral posterior shoulder dislocations with reverse Hill-Sachs lesions. In Case 1, the lesions were managed using the McLaughlin procedure and the modified McLaughlin procedure. In Case 2, open reduction and internal fixation of the left shoulder failed, and reconstruction of the right humeral head using bone cement was subsequently performed.

## Case presentation

### Case 1

A 56-year-old man, farmer, with a history of asthma was receiving regular oral aminophylline treatment. He was admitted to our hospital on June 24, 2024, due to bilateral shoulder pain and functional activity limitation that had persisted for nearly two months. Approximately 50 days earlier, he experienced a sudden syncopal episode while rising from a chair at home, resulting in a fall and transient loss of consciousness with witnesses. Upon regaining consciousness, the patient reported severe bilateral shoulder pain and restricted range of motion. He sought a medical evaluation at a local hospital, where he underwent a cranial computed tomography (CT) and anteroposterior X-rays of both shoulders. He was informed that there were no significant abnormalities, and he was discharged with conservative management, but the pain and functional impairment did not subside. Approximately 50 days, he presented to our hospital, where a CT scan revealed bilateral posterior shoulder dislocations, and he was admitted for further management.

**Fig. 1:**
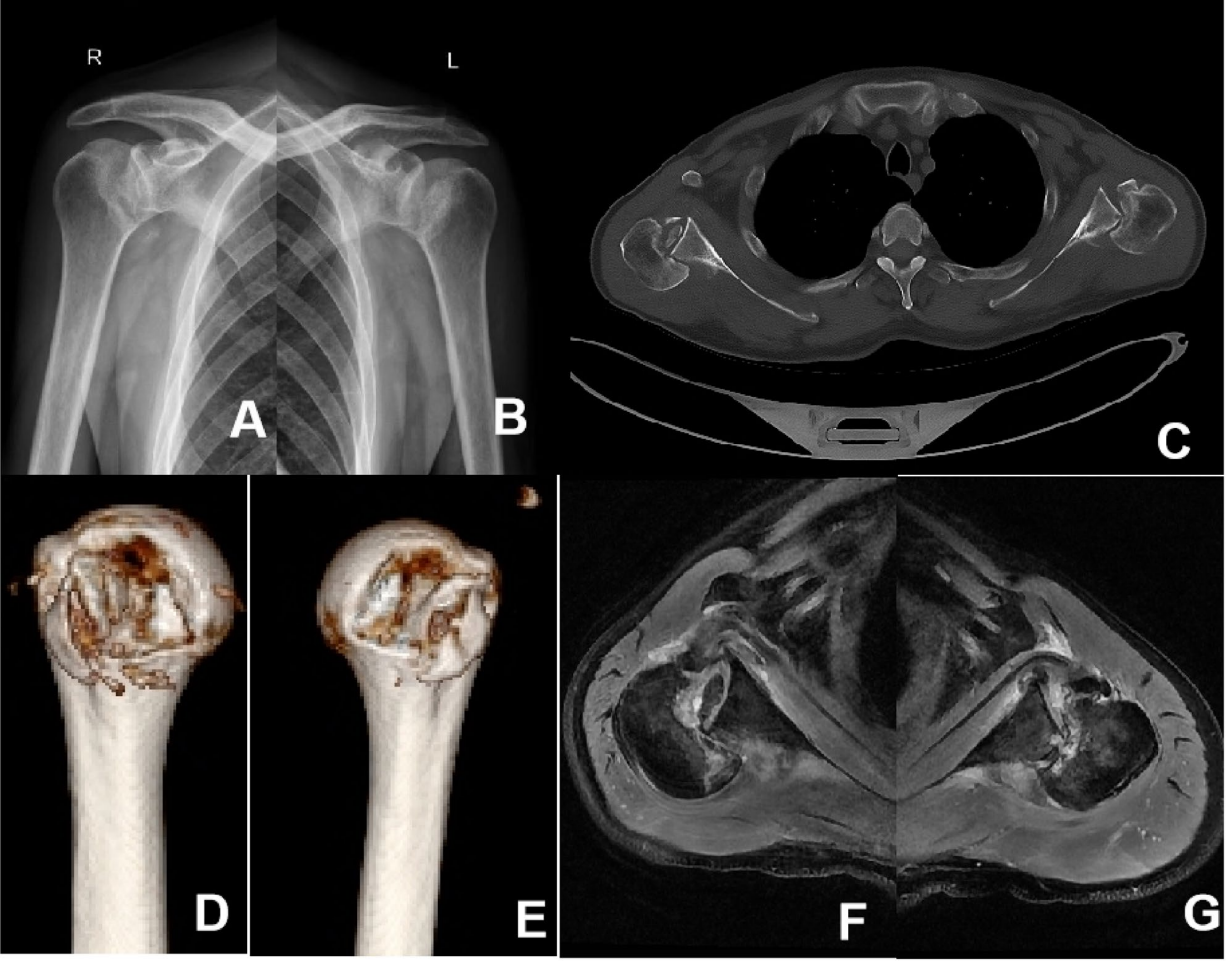
Preoperative images of the patient **A** AP view of the right shoulder; **B** AP view of the left shoulder; **C** Bilateral CT scans; **D** Three-dimensional reconstruction of the right humeral head; **E** Three-dimensional reconstruction of the left humeral head; **F** Right shoulder MRI scan; **G** Left shoulder MRI scan

Upon admission, the patient was alert. A physical examination indicated fixed internal rotation of both shoulder joints and significant tenderness in the shoulder area. Active range of motion, including flexion, extension, and abduction, was severely limited. The Dugas sign was absent, and sensation and distal circulation in both upper limbs were normal. Anteroposterior radiographs of both shoulders revealed a loss of joint space between the medial margin of the humeral head and the glenoid (Fig. 1A and B). Three-dimensional CT scans confirmed bilateral posterior dislocations of the humeral heads, which were locked against the glenoid, accompanied by substantial bone loss on the anteromedial aspect of the humeral heads (Fig. 1C-E). MRI of the shoulder joints showed posterior dislocations of both shoulders, injury of the subscapularis muscle, and a fracture of the lesser tuberosity of the right humerus (Fig. 1F-G). Cranial CT and MRI examinations revealed no abnormalities.

**Fig. 2:**
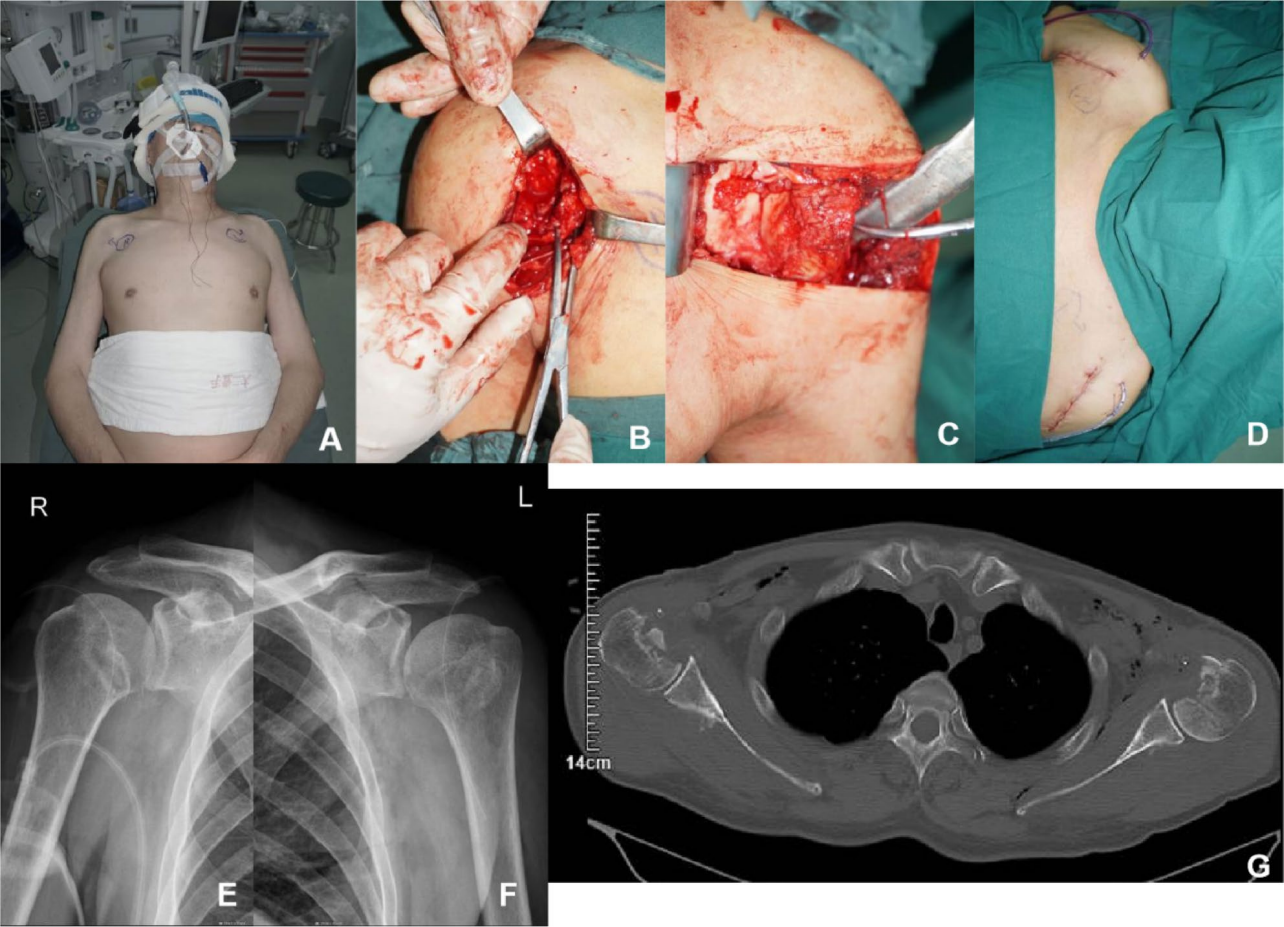
Intraoperative and postoperative images of Case 1. **A** Beach-chair position; **B** Articular surface defect of the right humeral head; **C** Articular surface defect of the left humeral head; **D** Incisions of both shoulders; **E** Postoperative AP view of the right shoulder; **F** Postoperative AP view of the left shoulder; **G** Postoperative CT scans of both shoulders

The patient was diagnosed with bilateral posterior shoulder dislocations with reverse Hill-Sachs lesions and bilateral rotator cuff injuries. Given the chronic nature of the dislocations and extensive bone defects exceeding 40% on both humeral heads, manual reduction was not attempted. Surgical intervention was performed under general anesthesia on June 26, 2024. The patient was positioned in a beach-chair position (Fig. 2A). The deltopectoral approach was used. On the right side, the fracture fragment of the lesser tuberosity and its attached subscapularis muscle were mobilized medially. On the left side, the subscapularis was detached at its insertion on the lesser tuberosity and retracted medially. The joint capsules were exposed and incised, revealing posterior dislocations of the humeral heads locked against the posterior glenoid. The humeral heads were unlocked by posterolateral traction, and reduction was achieved with external rotation aided by anelevator. Examination revealed compression defects on the anteromedial joint surfaces of the humeral heads, consistent with reverse Hill-Sachs lesions (Fig. 2B and C). On the right side, a modified McLaughlin procedure was performed. Two all-suture anchors were inserted at the medial edge of the humeral head defect. Sutures were passed through the junction of the lesser tuberosity and the subscapularis muscle. Two lateral-row anchors were placed to create a suture bridge, securing the fractured fragment of the lesser tuberosity. On the left side, the McLaughlin procedure was applied, with three all-suture anchors used to reattach the subscapularis tendon to the defect site. Both shoulder joints were assessed for stability, and no signs of recurrent dislocation were detected. A drainage tube was inserted, and the incisions were closed (Fig. 2D).

Postoperatively, measures for pain control and infection prevention were implemented. Active shoulder movement was restricted for four weeks, but flexion and extension of the hands, wrists, and elbows were encouraged. Follow-up X-rays and CT scans showed satisfactory reduction and alignment of the fractures (Fig. 2E-G). The6-month postoperative follow-up, a satisfactory functional result was achieved. He had two stable shoulders, a near-normal range of motion, and a Constant score of 92.

### Case 2

**Fig. 3:**
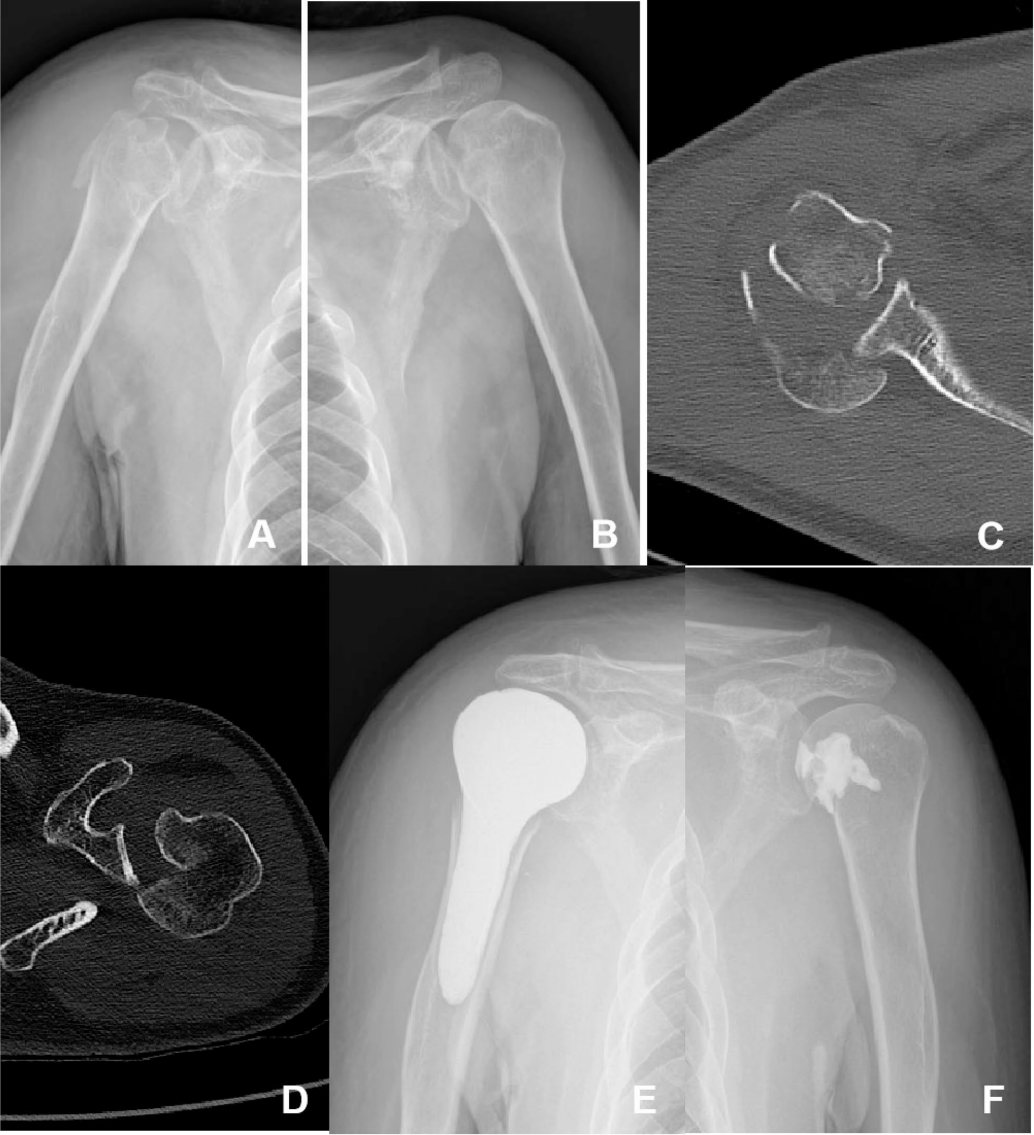
Images of Case 2. **A** AP view of the right shoulder; **B** AP view of the left shoulder; **C** CT scan of the right shoulder; **D** CT scan of the left shoulder; **E** Postoperative AP view of the right shoulder; **F** Postoperative AP view of the left shoulder

A 75-year-old man, farmer, with a history of epilepsy was admitted to our hospital on September 27, 2022, complaining of bilateral shoulder pain and restricted mobility following an epileptic seizure. Upon physical examination, the patient was alert, with both shoulders fixed in internal rotation, significant peri-shoulder tenderness, and limited active flexion, extension, and abduction. Sensation and distal circulation in both upper limbs were normal. Anteroposterior radiographs of the shoulders revealed a comminuted proximal right humeral fracture, an anterior fracture of the left humeral head, and a “light bulb sign” (indicating alignment of the humeral head with the shaft due to fixed internal rotation) in both shoulder joints (Fig. 3A and B). Three-dimensional CT scans showed bilateral posterior humeral head dislocations, a comminuted proximal right humeral fracture, and a large anteromedial bone defect in the left humeral head (Fig. 3C-E).

The patient was diagnosed with bilateral posterior shoulder dislocations and a comminuted proximal humeral fracture on the right side, along with a reverse Hill-Sachs lesion on the left side. Surgical intervention was performed under general anesthesia on October 1, 2022. Both shoulders were positioned in the beach-chair position, and a deltopectoral approach was utilized. On the right side, after joint reduction, an attempt was made to perform open reduction and internal fixation (ORIF) for the proximal humeral fracture. However, during the operation, it became apparent that the degree of comminution of the proximal humerus had been underestimated, and the proximal humerus could not be stabilized with a plate. As no shoulder prosthesis was prepared for the operation and considering the patient’s advanced age and low functional shoulder requirements, it was decided to resect the fracture fragments and reconstruct the proximal humerus morphology using bone cement, as well as reattach the rotator cuff tendons to the cement by drilling holes into it (Fig. 3F and G). Intraoperative testing of shoulder mobility confirmed that the cemented reconstruction and glenoid joint were stable. On the left side, after joint reduction, an anterior compressive fracture of the humeral head was identified. Reduction was performed using a lifting technique, and the bone defect was filled with bone cement. 1 year postoperatively, the range of motion on the right side was 70° of forward flexion, 60° of abduction, 50° of internal rotation, and 40° of external rotation. The range of motion on the left side was 100° of forward flexion, 85° of abduction, 80° of internal rotation, and 65° of external rotation. The Visual Analog Scale (VAS) score was 4, and Constant scores were 62 on the right side and 84 on the left side.

## Discussion and conclusions

A search of PubMed, Medline, Embase, Cochrane, Scopus and Google Scholar databases using various combinations of the keywords ‘shoulder’, ‘dislocation’, ‘posterior instability’, ‘reversed Hill Sachs’, ‘bone loss’, ‘reversed bony bankart’, ‘osseous glenoid defects’, ‘glenoid bone grafting’, ‘glenoid’,‘humeral head’, ‘glenohumeral’, and ‘capsulolabral’ was performed to include articles that were published between 2000 and 2024. 342 articles were evaluated, 318 articles were excluded due to ‘anterior shoulder dislocation’, ‘bony bankart’ and ‘shoulder dislocation without fracture’, and finally 24 papers were considered for the review.

Posterior shoulder dislocation is relatively rare in clinical practice, accounting for only 2–4% of all shoulder dislocations [1]. The primary causes include seizures, electrical injuries, withdrawal from drugs or alcohol, falls from heights, and motor vehicle accidents, with young adult males being the most affected [2]. At the onset, generalized muscle spasms cause the internal rotators and adductors to overpower the external rotators and abductors, resulting in extreme internal rotation of the upper limb. This leads to the humeral head dislocating posteriorly, breaching the posterior glenoid rim and joint capsule [3]. Despite the characteristic limitation of external rotation following posterior dislocation, some degree of abduction and internal rotation may persist. In some instances, the “Dugas sign” is considered negative, which may misleadingly suggest the absence of shoulder dislocation. When combined with the subtle presentation on standard anteroposterior radiographs, the misdiagnosis rate can reach 60–79% [4–6]. To reduce misdiagnosis in high-risk populations, such as those with seizures, electrical injuries, or high-energy trauma, supplementary imaging techniques including scapular lateral views, axillary views, CT scans, or MRI are recommended. Posterior shoulder dislocations frequently lead to complications due to persistent muscle spasms, such as impaction of the anteromedial articular surface of the humeral head against the posterior glenoid. This can result in compression fractures of the anteromedial humeral head (reverse Hill-Sachs lesions), fractures of the lesser tuberosity, posterior labral tears, and a locked engagement between the humeral head and the glenoid. These complications occur in approximately 86% of posterior humeral head dislocations [4]. Bilateral posterior shoulder dislocations combined with reverse Hill-Sachs lesions are infrequently reported in the literature. In Case 1, the injury mechanism involved syncope, and there were no witnesses present during the event. The patient regained consciousness spontaneously; however, the exact injury mechanism remains unclear. To further investigate the cause of syncope, cranial CT scans were performed, which revealed no abnormalities, and the patient had no relevant medical history. The diagnosis of epilepsy could not be confirmed as the patient could not clearly recall the pre-syncope experience. The syncopal episode was hypothesized to involve epileptic-like muscle contractions. In Case 2, the patient had a documented history of epilepsy.

The treatment of posterior shoulder dislocation with reverse Hill-Sachs lesions depends on the size of the humeral head defect, the duration of the dislocation, the patient’s functional requirements, and the stability of the shoulder joint [7, 8]. The Cicak method is the most commonly used technique for assessing the size of defects [9]. It involves selecting the axial CT image of the shoulder that displays the largest humeral head defect. The articular surface is then divided into four equal parts along the anterior-posterior line, with each segment representing 25%. Defect size is classified as mild if it is less than 25%, moderate if it ranges from 25% to 50%, or large if it exceeds 50%.

Another method for evaluating defect size is the Moroder method [10]. This approach involves drawing a virtual circle that fits the humeral head on the axial CT image that shows the largest defect. The center of the circle is connected to the midpoint of the intertubercular groove (referred to as line A), the anterior and posterior edges of the defect (lines B and C), and the posterior rim of the glenoid (line D). The angles formed by these lines describe the characteristics of the defect: the α angle (between lines B and C) indicates the size of the defect, the β angle (between lines A and B) reflects the location of the defect, and the Δ angle (between lines C and D) indicates the range of internal rotation until the humeral head locks against the posterior glenoid. The γ angle, which is the sum of the αand β angles, offers a comprehensive description of the size and location of reverse Hill-Sachs lesions.

Injuries less than three weeks old with defect sizes under 25% should be treated with closed reduction under general anesthesia [11, 12]. Following a successful reduction, the shoulder should be immobilized in a slightly abducted and externally rotated position for a duration of 4–6 weeks. For injuries older than three weeks or those with larger defect sizes, surgical intervention is necessary. Given the low incidence of posterior shoulder dislocations accompanied by reverse Hill-Sachs lesions, there is a lack of large-scale, evidence-based research to determine the optimal surgical approach. The main surgical options are as follows: (1) Humeral Head Reconstruction. This approach entails elevating the collapsed area of the humeral head and filling the defect with bone graft or bone cement to restore structural integrity, and is suitable for young patients with good bone quality and short dislocation durations [1, 8, 13]. (2) Tendon Transfer: The McLaughlin procedure entails securing the subscapularis muscle’s insertion to the site of the defect following reduction of the glenohumeral joint. The modified McLaughlin procedure includes an osteotomy of the lesser tuberosity, followed by the attachment of the tuberosity, along with the attached subscapularis, to the defect. Rinaldi et al. [14] reported that the modified McLaughlin procedure is the most popular for defects less than 50%. In this case, the left humeral head defect was approximately 40%, without a lesser tuberosity fracture; the subscapularis was detached at its insertion and reconstructed at the defect site using suture anchors. The right humeral head defect was approximately 50%, accompanied by a lesser tuberosity fracture. The lesser tuberosity was mobilized and fixed into the defect after reduction. (3) Shoulder Arthroplasty: Total or hemi-shoulder arthroplasty may be performed in patients with significant defects, chronic dislocations, or humeral head necrosis. Although it is effective in alleviating pain and enhancing function, arthroplasty should be carefully considered for young patients with high functional demands, as it is a definitive treatment [12, 15]. (4) Arthroscopy: Advances in arthroscopic techniques now allow the McLaughlin and modified McLaughlin procedures to be performed arthroscopically [16]. Arthroscopy enables a precise evaluation of humeral head defects, assisted closed reduction, removal of loose fragments, and repair of labral tears. It is particularly advantageous for patients with smaller reverse Hill-Sachs lesions [17, 18]. However, for larger defects requiring procedures such as allograft bone reconstruction, open surgery remains more appropriate. In this case, we used bone cement to reconstruct the proximal humerus morphology as a remedial salvage measure. Compared with the articular prostheses, the surface of the bone cement prosthesis is not smooth and flat, and the mobility of the shoulder joint will be limited after the operation. It may also be a great challange partial obstruction if subsequent shoulder arthroplasty or revision surgury is performed. Compared with anatomic shoulder arthroplasty, a cemented proximal humeral hemi-arthroplasty presents a deliberately rough surface that cannot congruently match the glenoid fossa, and the rotator cuff has no biological capacity to heal to polymethyl-methacrylate. Over time this incongruity and lack of cuff integration lead to eccentric glenoid wear, turning any future revision or conversion to total shoulder replacement into a considerably more demanding procedure. Nevertheless, we still regard this technique as a salvage option after failed open reduction and internal fixation of proximal humeral fractures, particularly in low-demand elderly patients in whom glenohumeral function is a secondary priority.

In summary, posterior shoulder dislocation with reverse Hill-Sachs lesions is prone to misdiagnosis. With increased understanding and awareness of this condition, the rate of misdiagnosis is expected to decrease significantly. Early diagnosis and appropriate treatment, based on the severity of the injury, are necessary for a good functional outcome. For particularly comminuted proximal humeral fractures, when internal fixation is not possible and no joint replacement prosthesis is available, bone cement reconstruction is an option.

## Data Availability

All data generated or analyzed during this study are included in this published article.

## Abbreviations

CT: Computed Tomography
MRI: Magnetic Resonance Imaging
ORIF: Open Reduction and Internal Fixation
ROM: Range of Motion
